# Correction to “Evaluation of the Antiwear‐Ability/Scratch‐Resistance Efficacy of Makeup Products by In Vitro Test Method Application”

**DOI:** 10.1111/srt.70072

**Published:** 2025-08-08

Jiang Y, Xu Z, Qiu Y, Chen G, Wang S. Evaluation of the antiwear‐ability/scratch‐resistance efficacy of makeup products by in vitro test method application. *Skin Res Technol*. 2023;29:e13420. doi:10.1111/srt.13420

In the article, there were errors in References 5 to 11.

The incorrect references are:

5. Williams LN, Coles LR. Consumer perceptions of Foundation makeup product efficacy. *Int J Cosmet Sci*. 2019;**41**(5):416‐424.

6. Xu J, Giddens SE, Sivamani R. Evaluating the performance of liquid Foundation makeup on aging facial skin using a 3D skin imaging system. *J Cosmet Dermatol*. 2019;**18**(4):1054‐1061.

7. Fukayama K, Ikoma H, Sakai K, et al. Comparison of two in vivo methods for evaluating facial Foundation makeup. *J Oleo Sci*. 2019;**68**(1):13‐18.

8. Souza MA, Campos MM, Arruda TP, et al. In vivo and in vitro evaluation of transfer‐resistant properties of Foundation makeup. *Skin Res Technol*. 2020;**26**(3):296‐303.

9. Pollitt K, Heusel L, McAtamney R. Development of an in vivo and in vitro method for evaluating the efficacy of long‐wearing Foundation makeup. *Int J Cosmet Sci*. 2021;**43**(1):9‐16.

10. Weschler CJ, Worsley C, Nazaroff WW. Using makeup Foundation to assess chemical exposure from air and dust: differences by product type and formulation. *Environ Sci Technol*. 2019;**53**(2):763‐771. doi:10.1021/acs.est.8b05338

11. van der Kolk MM, de Lima Carvalho L, Weijland J, Verboven MD, van der Molen AM. Measuring the amount of moisturizing cream applied on human skin: an ex vivo method. *Skin Pharmacol Physiol*. 2016;**29**(1):19‐25. doi:10.1159/000441984

The correct references are:

5. Yan Y, Lee J, Hong J, et al. Measuring and describing the discoloration of liquid foundation. *Color Res Appl*, 2020. doi:10.1002/col.22584

6. Keita S, Ikuyo S. Evaluation method of makeup collapse and evaluation model of makeup collapse: JP 2019001738 A[P]. 2019‐01‐10.

7. Jessica A, Joseph B, Mehdi D. Quantitative evaluation for transfer resistance of face foundation products: fast screening (in‐vitro) vs. consumer‐centric (in‐vivo) assessment. *Proceedings of the 26th IFSCC Conference “Cosmetic Science that Awakens the Senses”*. 2021:2668‐2671.

8. Badami JV, Bui HS Quantification of the color transfer from long‐wear face foundation products: The relevance of wettability. In: Mittal KL, Bui HS, eds. *Surface Science and Adhesion in Cosmetics*. 2021. doi:10.1002/9781119654926.ch12.

9. ASTM International. Standard Test Method for Evaluation of Scratch Resistance of Polymeric Coatings and Plastics Using an Instrumented Scratch Machine: ASTM D7027‐13 [S]. 2013. doi:10.1520/d7027-13.

10. Robert P. Container e.g. for cosmetic product has scratch‐resistant plastic cover for at least part of outer surface: FR 2799182 A1[P]. 2001‐04‐06.

11. Alam S, Algahtani MS, Ahmad MZ, et al. Investigation utilizing the HLB concept for the development of moisturizing cream and lotion: In‐vitro characterization and stability evaluation. *Cosmetics*. 2020;7(2):43. doi:10.3390/cosmetics7020043.

We apologize for these errors.

